# 

**Published:** 1905-11-04

**Authors:** 


### NOTICE TO CORRESPONDENTS.

All MSS., Letters, Books for Review, and other matters intended for the Editor should be addressed The Editor, "The Hospital," The Hospital Buildings, 28 & 29 Southampton Street, Strand, W.C.

The Editor cannot undertake to return rejected MS., even when accompanied by stamped directed envelope.

**Notice to Advertisers and Subscribers.**

All Advertisements, Orders for Copies of The Hospital, and Business Communications should be addressed to **THE MANAGER (Not to the Editor),** The Hospital, 28 & 29 Southampton Street, Strand London, W.C.

The Cost of Subscription, post free, is at follows:—Per week, 3½d.; per quarter, 3s. 3d.; per half-year, 6s. 6d.; per annum, 13s. Foreign and Colonial:—Per annum, 19s. 6d., payable, in all cases, in advance.

**NOTICE.—Vol. XXXVII. of "The Hospital" October 1904 to March 1905), in handsome cloth binding, will shortly be on sale at the office of this Journal, price 10s. 6d. Cases for binding the Numbers contained in this Volume 2s. Index to Vol. XXXVII., 3½d., post free.**

**The Monthly Part for November is now ready. Price 1s., by post, 1s. 3d.**

### BOOKS RECEIVED.

Cassell and Co.

"Materia Medica and Therapeutics." By J. Mitchell Bruce, M.D.

W. H. and L. Collingridge.

"Dairy Cows and the Dairy."

David Bryce and Son.

"A System of Surgical Nursing." By A. N. M'Gregor, M.D.

Simpkin, Marshall and Co.

"Text-book of Hygiene based on Physiology for School Teachers." By A. Watt Smyth.

Cassell and Co.

"Organotherapy." By H. Batty Shaw, F.R.C.P.

Hutchinson and Co.

"The Mother's Manual." By E. L. Coolidge, M.D.

"Beauty through Hygiene." By E. E. Walker, M.D.

W. Heinemann.

"Medicine and the Public." By S. Squire Sprigge, M.D.

Longmans, Green, and Co.

"Notter and Firth's Hygiene." Sixth edition.

"Pall Mall" Press.

"The Doctor and the Simple Life." By C. W. Saleeby, M.D.

S. W. Partridge and Co.

"The Zenana, or Woman's Work in India." Vol. 12, 1905.

### MEDICAL APPOINTMENTS.

**Alford, E. Frank R.,** M.R.C.S.Eng., L.R.C.P.Lond., House Physician at the City of London Hospital for Diseases of the Chest, Victoria Park, London, E. **Ash, B. M.,** Assistant House Surgeon at the Salisbury Infirmary. **Bathurst, Lacey,** M.B., B.S., L.R.C.P.Lond., M.R.C.S., Senior House Surgeon to the Croydon General Hospital. **Davis, Harry,** L.R.C.P.Lond., M.R.C.S., L.S.A., D.P.H.Cantab., Medical Officer of Health for the Callington Urban District (Cornwall) for three years. **Graham, Lewis,** L.R.C.P.Lond., M.R.C.S., Junior House Surgeon to the Croydon General Hospital. **Henderson, Thomas Bonhote,** M.B., B.Ch.Oxon., House Surgeon at the Salisbury Infirmary. **Hummel, J. J.,** M.Sc., Resident Clinical Assistant to the Leeds Hospital for Women and Children. **Kerr, James R.,** M.B., Ch.B.Glasg., House Surgeon to the Royal Hospital for Sick Children, Glasgow. **Leach, H.,** M.R.C.S., L.R.C.P.Lond., House Surgeon at the Leeds Hospital for Women and Children. **McElwaine, Thomas,** L.R.C.P. & S.Edin., L.F.P.S.Glasg., Public Vaccinator for the Stoke District by the Devonport Board of Guardians. **Saunders, Ernest George Symes,** M.D., C.M.Aberd., Assistant Surgeon to the Royal Albert Hospital, Devonport. **Scott, T. Graham,** M.R.C.S., L.R.C.P.Lond., House Anæsthetist at the Royal Dental Hospital, London. **Turner, J. G.,** F.R.C.S., L.R.C.P., L.D.S., Lecturer on Dental Surgery and Pathology to the Royal Dental Hospital, London. **Wood, C. G. Russ,** F.R.C.S.Eng., L.R.C.P.Lond., Honorary Ophthalmic and Aural Surgeon to the Wrexham Infirmary.

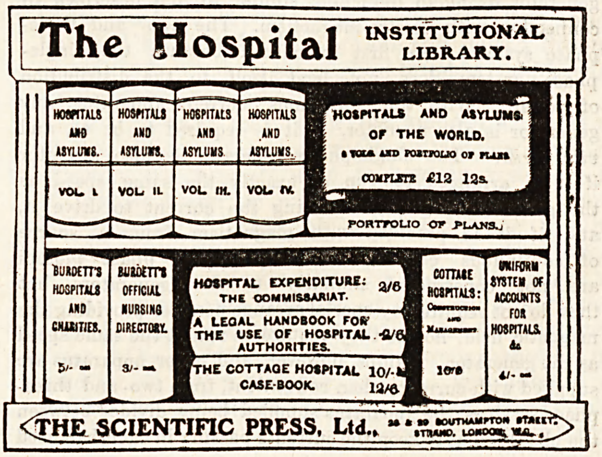

### Books for

MATRONS

SISTERS

HOSPITAL NURSES

PROBATIONERS

PRIVATE NURSES

**Cottage Hospital.** General, Fever, and Convalescent. Their Construction and Management. By Sir Henry Burdett. **10s. 6d.** post free.

**Furnishing and Appliances of a Cottage Hospital.** An Actual Inventory. **6d.** net; **7d.** post free.

**Hospital Expenditure:** The Commissariat. **2s. 6d.** post free.

**Cottage Hospital Case-Book of Register of Patients.** No. 1, **10s. 3d.** post free. No. 2, **12s. 10d.** post free.

**Hospital Sisters and Their Duties.** By Eva E. C. Lückes, Matron London Hospital. **2s. 6d.** post free.

**A Handbook for Nurses.** By Dr. J. K. Watson. **5s.** net.

**Nursing in Diseases of the Throat, Nose, and Ear.** By Macleod Yearsley. **2s. 6d.** post free.

**Elementary Treatise on the Light Treatment.** By James H. Sequeria, M.D. **2s. 6d.** net; **2s. 9d.** post free.

**Medical Electricity and Light Treatment.** By Sister Kate Neale. Illustrated, cloth, **2s. 6d.** net; **2s. 9d.** post free.

**How to Become a Nurse: How and Where to Train.** By Sir Henry Burdett, K.C.B. **2s.** net; **2s. 4d.** post free

**Nursing Hints to Probationers on Practical Work.** The duties of a Nurse fully explained. By Annesley Voysey. **2s.** net; **2s. 3d.** post free.

**Art of Massage.** By Mrs. Hale. **6s.** post free.

**Pharmacy and Dispensing for Nurses.** By C. J. S. Thompson. **1s.** post free

**Gynæcological Nursing.** By Dr. Hawkins Ambler. **1s.** post free.

**Modern Nursing in Private Practice.** By Sir William Bennett. **6d.** net, **7d.** post free.

**Pocket Book of Temperature Charts.** Containing 17 Morning and Evening and 8 Four Hour Charts. **6d.** net, **7d.** post free.

**Examination of the Urine.** By J. K. Watson, M.D. **1s.** net, **1s. 1d.** post free.

**Linen Register for Large and Small Hospitals.** **7s. 6d.** net; **7s. 10d.** post free.

**Syllabus of Lectures to Nurses.** By Dr. Davidson. **1s.** post free.

**Temperature Charts.** Morning and Evening and Four Hour Charts. Size 10 in. by 8 in.

25, **1s.** 50, **1s. 6d.** 100, **2s. 9d.** | 250, **6s. 6d.** 500, **12s.** 1,000, **23s.**

Carriage paid.

**Japanned Tin Washable Chart Holders.** Specially designed for above Charts, each, **1s.** post free, per dozen, **9s. 6d.**

**Ministering Women.** The Story of the Royal National Pension Fund. **2s. 6d.** post free.

**Surgical Ward Work and Nursing.** By Dr. Miles. **3s. 6d.** net; **3s. 10d.** post free.

**Ophthalmic Nursing.** By Sydney Stephenson, M.B., F.R.C.S.E. **3s. 6d.** net; **3s. 10d.** post free.

**Nurses' Pronouncing Dictionary of Medical Terms and Nursing Treatment.** **2s.** post free.

**Nursing of Sick Children.** By Dr. Burnet. Cloth, **1s.** net, **1s. 1d.** post free.

**Elementary Anatomy and Surgery for Nurses.** By Dr. McAdam Eccles. **2s. 6d.** post free.

**Simple Sanitation.** The Practical Application of the Laws of Health to Small Dwellings. By Miss M. Loane. **1s.** net, **1s. 2d.** post free.

**Modern Nursing of Consumption.** By Dr. Jane Walker. **1s.** net, **1s. 2d.** post free.

**Case-Book for Private Nursing.** Size of an Exercise Book. 48 pp. **3d.** net, **4½d.** post free.

**Elementary Physiology for Nurses.** By C. F. Marshall, M.D. **2s.** post free.

**Practical Guide to Surgical Bandaging and Dressings.** By Dr. Johnson Smith. **2s.** post free.

**The Scientific Press, Ltd.**

Publishers of Nursing Text-Books, Charts, and Case-Books of all Descriptions.

28 & 29 SOUTHAMPTON STREET, STRAND, LONDON, W.C.

